# Supplementary material for: SESS Model for Adolescent Sexual Health Promotion: A Quasi-Experimental Two-School Evaluation in Thailand
Source: Int J Environ Res Public Health. 2025 Oct 8;22(10):1536. doi: 10.3390/ijerph22101536 (PMC12564106; doi:10.3390/ijerph22101536)
Supplement: Supplementary file 1 [file ijerph-22-01536-s001.zip › ijerph-3815335-supplementary.pdf]

Supplementary

Key Performance Indicators (KPIs) used for process evaluation of the SESS model. Indicators reflect dose delivered versus planned, facilitator fidelity, and student engagement across classroom and online components. Attendance and fidelity data were drawn from facilitator checklists and observation records, while digital engagement metrics were obtained from Facebook and LINE platform analytics. Minor adaptations (e.g., session rescheduling due to exams, shortened activities, reduced online activity after week 8) are noted to support transparency in program implementation.

Table S1. Key Performance Indicators (KPIs) for Process Evaluation of the SESS Model

KPI Category	Indicator	Planned	Delivered /Observed	% Achieved	Notes/ Adaptations
**Dose**	Number of sessions scheduled	16	16	100%	One session rescheduled due to exams; all sessions completed within the study period
	Average duration per session (minutes)	90	85	94.4%	Some sessions shortened to accommodate school activities and time constraints
	Average student attendance per session (%)	100%	92 %%	___%	• Absences mainly due to exam schedules, illness, and extracurricular commitments; attendance particularly lower during weeks 10–12
**Fidelity**	Facilitator adherence checklist (items)	20	19	95%	Role-play activities omitted in 2 sessions because of limited time
	Observed sessions with full delivery (%)	100%	100%	100%	All observed sessions delivered core components as intended
**Engagement**	Active users per week (Facebook group)	120	115	95.8%	Some students did not consistently log in despite being group members
	Median time on platform (minutes/week)	30	28	93.3%	Engagement declined slightly after week 8 as novelty decreased
	Average posts/comments per student	≥1	0.8	80%	Posting activity peaked in weeks 5–6 during group projects, then declined
